# Multi-group analysis using generalized additive kernel canonical correlation analysis

**DOI:** 10.1038/s41598-020-69575-x

**Published:** 2020-07-28

**Authors:** Eunseong Bae, Ji-Won Hur, Jinyoung Kim, Jun Soo Kwon, Jongho Lee, Sang-Hun Lee, Chae Young Lim

**Affiliations:** 1 Department of Statistics, University of California, Davis, CA USA; 2 Department of Psychology, Korea University, Seoul, Korea; 3 Department of Brain & Cognitive Sciences, Seoul National University, Seoul, Korea; 4 Department of Psychiatry, Seoul National University, Seoul, Korea; 5 Department of Electrical and Computer Engineering, Seoul National University, Seoul, Korea; 6 Department of Statistics, Seoul National University, Seoul, Korea

**Keywords:** Mathematics and computing, Statistics

## Abstract

Multivariate analysis has been widely used and one of the popular multivariate analysis methods is canonical correlation analysis (CCA). CCA finds the linear combination in each group that maximizes the Pearson correlation. CCA has been extended to a kernel CCA for nonlinear relationships and generalized CCA that can consider more than two groups. We propose an extension of CCA that allows multi-group and nonlinear relationships in an additive fashion for a better interpretation, which we termed as Generalized Additive Kernel Canonical Correlation Analysis (GAKCCA). In addition to exploring multi-group relationship with nonlinear extension, GAKCCA can reveal contribution of variables in each group; which enables in-depth structural analysis. A simulation study shows that GAKCCA can distinguish a relationship between groups and whether they are correlated or not. We applied GAKCCA to real data on neurodevelopmental status, psychosocial factors, clinical problems as well as neurophysiological measures of individuals. As a result, it is shown that the neurophysiological domain has a statistically significant relationship with the neurodevelopmental domain and clinical domain, respectively, which was not revealed in the ordinary CCA.

## Introduction

Multivariate analysis is a statistical method that considers several variables simultaneously. Compared with univariate analysis, which focus on the influence of one variable only, multivariate analysis takes into account not only the effect of each variable but also interaction between variables. Thus, multivariate analysis gets popular as researchers face to more complex data. A number of statistical methods concerning multivariate analysis have been developed and widely used. For instance, principle component analysis (PCA), first proposed by Pearson^1^ is a method that compresses the data in the high dimensional space into the low dimensional space by identifying dimensions in which the variability of the data are explained the most. Factor analysis extracts underlying, but unobservable random quantities by assuming variables are expressed with those random quantities^2^.

One of the popular multivariate analysis is canonical correlation analysis (CCA). CCA, proposed by Hotelling^3^, explores association between two multivariate groups. CCA finds linear combinations of each group that maximize a Pearson correlation coefficient between them. In this way, CCA can also serve as a dimension reduction method as each multi-dimensional variable is reduced to a linear combination. This advantage makes CCA widely used in many scientific fields that mostly deal with high dimensional data such as psychology, neuroscience, medical science and image recognition^4–7^, etc.

Despite of its strength, CCA has some limitations. CCA is restricted to linear relationship only so that the result of CCA can be misleading if two variables are linked with a non-linear relation. This limitation is inherited from the characteristics of the Pearson correlation. For example, if two random variables *X* and *Y* are related with the equation $X^2+Y^2=1$, then the Pearson correlation of *X* and *Y* results in $\mathrm {Corr}(X,Y)=0$, although two random variables are related. To overcome the linearity constraint of the classical CCA, Bach and Jordan^8^ proposed Kernel canonical correlation analysis (KCCA), which applies a kernel method to the CCA problem. Unlike CCA, KCCA is a method of finding nonlinear relationship between two groups. Kernelization allows practical nonlinear extension of the CCA method. KCCA has been successful in some scientific fields that need to find nonlinear relationship beyond linear one such as speech communication science, genetics and pattern recognition^9–11^, etc.

Another limitation of the classical CCA is that it is only applicable to two groups. Often, scientific experiments yield results that can be divided into more than two groups. Pair-wise application of CCA into the groups more than two could ignore the connection and non-connection within the groups. Multi-group version of CCA to overcome such limitation was introduced by Kettenring^12^, named generalized canonical correlation analysis (GCCA or MCCA). GCCA finds linear combinations of each group that optimize certain criterion, such as the sum of covariances. Tenenhaus et al.^13^ proposed kernelized version of GCCA termed as kernel generalized canonical correlation analysis (KGCCA). This method is an extension of CCA by combining nonlinearity and multi-group analysis. In spite of fully flexible extension, kernelization of all variables together in each group is not helpful to provide structural analysis of variables. For instance, it is difficult to see the contribution of one variable, say, $X_{11}$ in a group ${{\textbf{X}}}_1 = (X_{11}, \ldots , X_{1p_1})^T$ in relation to the another group ${{\textbf{X}}}_2=(X_{21},\ldots , X_{2p_2})^T$ using KGCCA. Balakrishnan et al.^14^ considered an additive model by restricting possible non-linear functions to the class of additive models. This modification enables to analyze the contribution of each variable. However, it is still restricted to two groups.

In this paper, we consider an additivity idea with more than two groups. We call our proposed approach as *generalized additive kernel canonical correlation analysis* (GAKCCA). We expect the proposed approach has a better interpretability than KCCA or KGCCA and it can be applied to multi-group data. The proposed approach was motivated by a research problem on investigating the relationships among individual measures such as divergent psychological aspects mainly measured psychometric questionnaires and neurophysiological aspects such as brain morphologies. In this study, we analyze four domains of individual variables: neurodevelopmental, psychosocial, clinical characteristics, and structural MRI (Magnetic Resonance Image) measures. The present study was not only to define the link between the above four domains but also to reveal phase of variables of each domain under the hypothesis that a series of associations between domains are assumed to exist. We expect that the proposed method would facilitate identifying the link of neurophysiological basis represented by structural MRI related variables with the psychological variables.

The organization of the paper is as follows. In Materials and Methods section, we review CCA and its variants, then specify the population and empirical versions of the proposed GAKCCA method and introduce how to define the contribution of a variable in a group. As the proposed approach requires a regularization parameter, we discuss selection of a regularization parameter as well. Hypothesis test based on permutation is also performed. In Results section, we show the results of simulation study to confirm that our method is valid and it explains the relationship of groups well. The results of real data analysis are also presented here. Finally, the discussion is given in the “Discussion” section.

## Materials and methods

We first briefly review CCA and its variants. Then, we present our GAKCCA method and describe the algorithm for implementation.

### Canonical correlation analysis and its variants

For two multi-variate groups, canonical correlation analysis finds linear combination of each group that maximizes correlation between two linear combinations. That is, CCA finds ${{\mathbf{b}}}_1=(b_{11},\ldots , b_{1p_1})^T$ and ${{\mathbf{b}}}_2= (b_{21},\ldots , b_{2p_2})^T$ that satisfy the following: $\max _{{{\mathbf{b}}}_1, {{\mathbf{b}}}_2} \displaystyle \mathrm {Cov}\left( {{\mathbf{b}}}_1^T {{\mathbf{X}}}_1, {{\mathbf{b}}}_2^T {{\mathbf{X}}}_2 \right)$ subject to $\mathrm {Var}\left( {{\textbf{b}}}_1^T {{\textbf{X}}}_1 \right) = \mathrm {Var}\left( {{\textbf{b}}}_2^T {{\textbf{X}}}_2 \right) = 1$, where ${{\textbf{X}}}_1 = (X_{11}, \ldots , X_{1 p_1})^T$ has $p_1$ variables and ${{\textbf{X}}}_2 = (X_{21}, \ldots , X_{2 p_2} )^T$ has $p_2$ variables. Here, $(\cdot \,)^T$ denotes the transpose of a matrix. Variance constraints are to reduce the freedom of scaling for ${{\mathbf{b}}}_1$ and ${{\mathbf{b}}}_2$.

Instead of linear combination of variables in each group in CCA, Kernel canonical correlation analysis utilizes nonlinear functions to extract the relationship between two groups. KCCA can be formulated as follow: $\max _{f_1,f_2} \mathrm {Cov}\Big ( f_1({{\textbf{X}}}_1), f_2({{\textbf{X}}}_2) \Big )$ subject to $\mathrm {Var}\Big ( f_1({{\textbf{X}}}_1) \Big ) = \mathrm {Var}\Big ( f_2({{\textbf{X}}}_2) \Big ) = 1$, where $f_j : {\mathbb {R}}^{p_j} \rightarrow {\mathbb {R}}$ for $j=1,2$ is an unknown function in the reproducing kernel Hilbert space (RKHS)^8^.

Note that both CCA and KCCA assume two groups of variables. To expand beyond two groups, Kettenring^12^ introduced multi-group generalization of CCA (GCCA or MCCA). GCCA finds linear combinations of each group that optimize certain criterion to reveal multi-group structure. Given *J* multi-variate groups ${{\textbf{X}}}_1, \ldots , {{\textbf{X}}}_J$, GCCA finds ${{\textbf{b}}}_1, \ldots , {{\textbf{b}}}_J$ by considering $\max _{{{\textbf{b}}}_1, \ldots , {{\textbf{b}}}_J} \sum _{j,k=1;j \not =k}^{J} c_{jk} g \left[ \mathrm {Cov}\Big ( {{\textbf{b}}}_j^T {{\textbf{X}}}_j, {{\textbf{b}}}_k^T {{\textbf{X}}}_k \Big ) \right]$ subject to $\mathrm {Var}\Big ( {{\textbf{b}}}_j^T {{\textbf{X}}}_j \Big ) = 1 \hbox {~for~} j=1, \ldots , J.$ A function *g*, which is called a scheme function, is related to a criterion for selecting canonical variates^12^. The examples of *g* are $g(x) = x$ (Horst scheme^15^), $g(x)=|x|$ (Centroid scheme^16^) or $g(x)=x^2$ (Factorial scheme^17^). $c_{jk}$ is an element of $J \times J$ design matrix *C*, where $c_{jk} = 1$ if *j* and *k* groups are related and $c_{jk}=0$, otherwise.

Tenenhaus and Tenenhaus^18^ extended GCCA to a regularization version by imposing a constraint on the norm of a coefficient vector in a linear combination as well as the variance of the linear combination (RGCCA). Specifically, the constraint is given by $\tau _j ||{{\textbf{b}}}_j||^2 + (1- \tau _j) \mathrm {Var}\Big ( {{\textbf{b}}}_j^T {{\textbf{X}}}_j \Big ) = 1$ for $j=1, \ldots , J,$ where $\varvec{\tau } = (\tau _1, \ldots , \tau _J)^T$ is a regularization parameter vector (or shrinkage parameter). Regularization parameters enable an inversion operation by avoiding ill-conditioned variance matrices^13,18^. All $\tau _j$’s are between 0 and 1.

Also, Tenenhaus et al.^13^ developed a nonlinear version of GCCA (KGCCA) by considering a function for each group. That is, KGCCA finds $f_1,\ldots , f_J$ that satisfy $\max _{f_1,\ldots , f_J} \sum _{j,k=1;j \not =k}^{J} c_{jk} g \left[ \mathrm {Cov}\Big ( f_j({{\textbf{X}}}_j), f_k ({{\textbf{X}}}_k) \Big ) \right]$ subject to $\mathrm {Var}\Big ( f_j ({{\textbf{X}}}_j) \Big ) = 1 \hbox {~for~} j=1, \ldots , J,$ where each $f_j : {\mathbb {R}}^{p_j} \rightarrow {\mathbb {R}}$ is an unknown function in the RKHS. *g* and $c_{jk}$ are same as those in RGCCA.

### Generalized additive Kernel canonical correlation analysis

In this subsection, we introduce our approach that considers an additive structure in the multi-group setting. As in the previous subsection, we consider *J* multi-variate random variable groups ${{\textbf{X}}}_j = \left( X_{j1}, \ldots , X_{j p_j} \right) ^T \in {\mathbb {R}}^{p_j}$ for $j=1, \ldots , J$. KCCA considers a function on the *j*-th group variable, $f_j({{\textbf{X}}}_j)$, where $f_j$ is a nonlinear function in the RKHS. In our approach, GAKCCA, we assume that $f_j$ is an additive function in RKHS as in Balakrishnan et al.^14^. That is,

$$\begin{aligned} f_j \in {\mathscr {H}}_j = \left\{ h_j \text { }\Big | \text { } h_j (x_1, \ldots , x_{p_j} ) = \sum _{l=1}^{p_j} h_{jl} (x_{l}) \text { and } h_{jl} \in {\mathscr {H}}_{jl} \right\} , \end{aligned}$$

where each ${\mathscr {H}}_{jl}$ is an RKHS with a kernel $\phi _{jl} (\cdot ,\cdot )$. Then, GAKCCA finds $f_j \in {\mathscr {H}}_j$ that satisfies0

1 $$\begin{aligned} \max _{f_1, \ldots , f_J} \displaystyle \sum _{j,k=1 ; j \not = k}^{J} c_{jk} g \Big [ \mathrm {Cov}\Big ( f_j({{\textbf{X}}}_j), f_k({{\textbf{X}}}_k) \Big ) \Big ] \,\,\,\hbox {subject to}\, \mathrm {Var}\Big ( f_j({{\textbf{X}}}_j) \Big ) = 1 \,\hbox { for }\, j=1, \ldots , J, \end{aligned}$$

where *g* and $c_{jk}$ are a scheme function and an element of the design matrix *C*, respectively. Since we assume $f_j \in {\mathscr {H}}_j$, we can write $f_j ({{\textbf{X}}}_j) = \sum _{l=1}^{p_j} f_{jl}(X_{jl})$ so that (1) becomes

2 $$\begin{aligned} \max _{f_{11}, \ldots , f_{1 p_1}, \ldots , f_{J 1}, \ldots , f_{J p_J}} \displaystyle \sum _{j,k=1 ; j \not = k}^{J} c_{jk} g \left[ \sum _{l=1}^{p_j} \sum _{m=1}^{p_k} \mathrm {Cov}\Big ( f_{jl} (X_{jl}), f_{km} (X_{km}) \Big ) \right] \end{aligned}$$

subject to $\sum _{l=1}^{p_j} \sum _{l'=1}^{p_j} \mathrm {Cov}\Big ( f_{jl} (X_{jl}) , f_{jl'} (X_{jl'}) \Big ) = 1$ for $j=1, \ldots , J$. We denote the expression in the Eq. (2) as $\rho _{{{\textbf{X}}}_1, \ldots , {{\textbf{X}}}_J}$.

When we introduce a covariance operator on the RKHS, mathematical treatment can be simpler^13,19,20^. The mean operator $m_{{\mathscr {H}}_{jl}}$ with respect to $X_{jl}$ is defined by

$$\begin{aligned} \langle f_{jl} , m_{{\mathscr {H}}_{jl}} \rangle _{{\mathscr {H}}_{jl}} = E \Big ( f_{jl} (X_{jl}) \Big ) = E \Big ( \langle f_{jl}, \phi _{jl} (\cdot , X_{jl} ) \rangle _{{\mathscr {H}}_{jl}} \Big ), \end{aligned}$$

where $\langle \cdot , \cdot \rangle _{{\mathscr {H}}_{jl}}$ is an inner product on ${\mathscr {H}}_{jl}$. The covariance operator $\Sigma _{jl,km}$ with respect to $X_{jl}$ and $X_{km}$ can be also defined as

$$\begin{aligned} \langle f_{jl}, \Sigma _{jl,km} f_{km} \rangle _{{\mathscr {H}}_{jl}}= & {} \mathrm {Cov}\Big ( f_{jl} (X_{jl}), f_{km} (X_{km}) \Big ) \\= & {} E \Big ( \langle f_{jl}, \phi _{jl} (\cdot , X_{jl}) - m_{{\mathscr {H}}_{jl}} \rangle _{{\mathscr {H}}_{jl}} \langle f_{km}, \phi _{km} (\cdot , X_{km}) - m_{{\mathscr {H}}_{km}} \rangle _{{\mathscr {H}}_{km}} \Big ). \end{aligned}$$

Then, the Eq. (2) can be expressed as

3 $$\begin{aligned} \rho _{{{\textbf{X}}}_1, \ldots , {{\textbf{X}}}_J} = \max _{f_{11}, \ldots , f_{1 p_1} \ldots f_{J 1} \ldots f_{J p_J}} \displaystyle \sum _{j,k=1 ; j \not = k}^{J} c_{jk} g \left[ \sum _{l=1}^{p_j} \sum _{m=1}^{p_k} \langle f_{jl}, \Sigma _{jl,km} f_{km} \rangle _{{\mathscr {H}}_{jl}} \right] \end{aligned}$$

subject to $\sum _{l=1}^{p_j} \sum _{l'=1}^{p_j} \langle f_{jl}, \Sigma _{jl,jl'} f_{jl'} \rangle _{{\mathscr {H}}_{jl}} = 1$ for $j=1, \ldots , J.$

Note that the Eq. (3) is a theoretical expression. We now explain how to derive an empirical version using samples. Suppose that we have *n* samples of $\{ {{\textbf{X}}}_1, \ldots , {{\textbf{X}}}_J \}$. The *i*-th sample of ${{\textbf{X}}}_j$ is denoted by ${{\textbf{x}}}_j^{(i)} = \left( x_{j1}^{(i)}, \ldots , x_{j p_j}^{(i)} \right)$. Fukumizu *et al*.^21^ suggested an estimated mean operator ${\widehat{m}}_{jl}$ and an estimated covariance operator ${\widehat{\Sigma }}_{jl,km}$ which satisfy the following properties:

$$\begin{aligned} \langle f_{jl} , {\widehat{m}}_{{\mathscr {H}}_{jl}} \rangle _{{\mathscr {H}}_{jl}} = {\widehat{E}} \Big ( f_{jl} (X_{jl}) \Big ) = \frac{1}{n} \sum _{i=1}^{n} \langle f_{jl}, \phi _{jl} (\;dot, x^{(i)}_{jl} ) \rangle _{{\mathscr {H}}_{jl}} = \left\langle f_{jl}, \frac{1}{n} \sum _{i=1}^{n} \phi _{jl} (\cdot , x^{(i)}_{jl} ) \right\rangle _{{\mathscr {H}}_{jl}} \end{aligned}$$

and

4 $$\begin{aligned} \langle f_{jl}, {\widehat{\Sigma }}_{jl,km} f_{km} \rangle _{{\mathscr {H}}_{jl}}= & {} {\widehat{\mathrm {Cov}}} \left( f_{jl} (X_{jl}), f_{km} (X_{km}) \right) = \frac{1}{n} \sum _{i=1}^{n} {\hat{f}}_{jl} (x_{jl}^{(i)}) {\hat{f}}_{km} (x_{km}^{(i)}), \end{aligned}$$

where $$ {\hat{f}}_{jl} (x_{jl}^{(i)}) = \langle f_{jl} , {\widehat{\phi }}_{jl}^{(i)} \rangle _{{\mathscr {H}}_{jl}}$$, $${\widehat{\phi }}_{jl}^{(i)} = \phi _{jl}^{(i)} - \frac{1}{n} \sum _{\xi =1}^{n} \phi _{jl}^{(\xi )} $$ and $$ \phi _{jl}^{(i)} = \phi _{jl} ( \cdot , x_{jl}^{(i)} )$$.

Bach and Jordan^8^ utilized the linear space spanned by ${\widehat{\phi }}_{jl}^{(1)}, \ldots , {\widehat{\phi }}_{jl}^{(n)}$ denoted by ${\mathscr {S}}_{jl}$ to write $f_{jl} = \sum _{i=1}^{n} a_{jl}^{(i)} {\widehat{\phi }}_{jl}^{(i)} + f_{jl}^{perp}$, where $a_{jl}^{(i)}$ is a coefficient corresponding to ${\widehat{\phi }}_{jl}^{(i)}$ which needs to be estimated and $f_{jl}^{perp}$ is orthogonal to ${\mathscr {S}}_{jl}$. With these facts, we can further simplify the Eq. (4) by introducing an $n \times n$ symmetric Gram matrix ${{\textbf{K}}}_{jl}$^22^ whose $(i,i')$-component is $({{\textbf{K}}}_{jl})_{(i,i')} = \phi _{jl} (X_{jl}^{(i)}, X_{jl}^{(i')}) = \langle \phi _{jl}^{(i)}, \phi _{jl}^{(i')} \rangle _{{\mathscr {H}}_{jl}}$. The centered ${{\textbf{K}}}_{jl}$ can be represented as $\widehat{{{\textbf{K}}}}_{jl} = \left( I_n - \frac{1}{n} J_n \right) ^T {{\textbf{K}}}_{jl} \left( I_n - \frac{1}{n} J_n \right)$, where $I_n$ is the $n \times n$ identity matrix and $J_n$ is the $n \times n$ matrix whose components are all ones, and its $(i,i')$-component is $(\widehat{{{\textbf{K}}}}_{jl})_{(i,i')} = \langle {\widehat{\phi }}_{jl}^{(i)}, {\widehat{\phi }}_{jl}^{(i')} \rangle _{{\mathscr {H}}_{jl}}.$ Then, using

5 $$\begin{aligned} {\hat{f}}_{jl} (x_{jl}^{(i)}) = \left\langle f_{jl} , {\widehat{\phi }}_{jl}^{(i)} \right\rangle _{{\mathscr {H}}_{jl}} = \left\langle \sum _{i'=1}^{n} a_{jl}^{(i')} {\widehat{\phi }}_{jl}^{(i')} + f_{jl}^{perp}, {\widehat{\phi }}_{jl}^{(i)} \right\rangle _{{\mathscr {H}}_{jl}} = \sum _{i'=1}^{n} a_{jl}^{(i')} \langle {\widehat{\phi }}_{jl}^{(i')}, {\widehat{\phi }}_{jl}^{(i)} \rangle _{{\mathscr {H}}_{jl}} = \sum _{i'=1}^{n} a_{jl}^{(i')} (\widehat{{{\textbf{K}}}}_{jl})_{(i',i)} , \end{aligned}$$

the Eq. (4) becomes

$$\begin{aligned} {\widehat{\mathrm {Cov}}} \left( f_{jl} (X_{jl}), f_{km} (X_{km}) \right)= & {} \frac{1}{n} \sum _{i=1}^{n} \sum _{i'=1}^{n} \sum _{i''=1}^{n} a_{jl}^{(i')} (\widehat{{{\textbf{K}}}}_{jl})_{(i',i)} (\widehat{{{\textbf{K}}}}_{km})_{(i,i'')} a_{km}^{(i'')} = \frac{1}{n} {{\textbf{a}}}_{jl}^T \widehat{{{\textbf{K}}}}_{jl}^T \widehat{{{\textbf{K}}}}_{km} {{\textbf{a}}}_{km}, \end{aligned}$$

where ${{\textbf{a}}}_{jl} = \left( a_{jl}^{(1)}, \ldots , a_{jl}^{(n)}\right) ^T$. The third equality in the Eq. (5) is due to the fact that $f_{jl}^{perp}$ is orthogonal to ${\mathscr {S}}_{jl}$. ${\mathscr {S}}_{jl}$ is the inner-product linear space generated by $\left\{ {\widehat{\phi }}_{jl}^{(1)}, \ldots , {\widehat{\phi }}_{jl}^{(n)} \right\}$ with inner product $\langle \cdot , \cdot \rangle _{{\mathscr {H}}_{jl}}$. This leads to $\langle f_{jl}^{perp}, {\widehat{\phi }}_{jl}^{(i)} \rangle _{{\mathscr {H}}_{jl}}=0$ for all $i=1,\ldots ,n$.

Note that the centered Gram matrix $\widehat{{{\textbf{K}}}}_{jl}$ is singular since the sum of rows or columns is zero. Thus, the constraint $\sum _{l=1}^{p_j} \sum _{l'=1}^{p_j} \langle f_{jl}, {\widehat{\Sigma }}_{jl,jl'} f_{jl'} \rangle = \sum _{l=1}^{p_j} \sum _{l'=1}^{p_j} \frac{1}{n} {{\textbf{a}}}_{jl}^T \widehat{{{\textbf{K}}}}_{jl}^T \widehat{{{\textbf{K}}}}_{jl'} {{\textbf{a}}}_{jl'} = 1$ does not provide a unique solution to our method. So, similar to the regularization approach for the KCCA method^8,13^, we use $\sum _{i=1}^{n} a_{jl}^{(i)} {\widehat{\phi }}_{jl}^{(i)}$ instead of $f_{jl}$ and introduce regularization parameters $\tau _j >0$ in the constraint conditions such as

6 $$\begin{aligned} \sum _{l=1}^{p_j} \sum _{l'=1}^{p_j} \left\langle \sum _{i=1}^{n} a_{jl}^{(i)} {\widehat{\phi }}_{jl}^{(i)}, \left\{ (1-\tau _j){\widehat{\Sigma }}_{jl,jl'} + \tau _j \mathrm {I}_{jl, jl'} \right\} \sum _{i=1}^{n} a_{jl'}^{(i)} {\widehat{\phi }}_{jl'}^{(i)} \right\rangle _{{\mathscr {H}}_{jl}} = 1, \qquad j=1, \ldots , J, \end{aligned}$$

where $\mathrm {I}_{jl, jl'}$ is an identity operator if $l = l'$ and a zero operator, otherwise. With the $\widehat{{{\textbf{K}}}}_{jl}$, the Eq. (6) can be rewritten as

$$\begin{aligned} (1-\tau _j) \sum _{l=1}^{p_j} \sum _{l'=1}^{p_j} \frac{1}{n} {{\textbf{a}}}_{jl}^T \widehat{{{\textbf{K}}}}_{jl}^T \widehat{{{\textbf{K}}}}_{jl'} {{\textbf{a}}}_{jl'} + \tau _j \sum _{l=1}^{p_j} {{\textbf{a}}}_{jl}^T \widehat{{{\textbf{K}}}}_{jl} {{\textbf{a}}}_{jl} = 1, \qquad j=1, \ldots , J. \end{aligned}$$

In summary, the empirical version of the Eq. (3) with regularization parameters is expressed as

7 $$\begin{aligned} {\widehat{\rho }}_{{{\textbf{X}}}_1, \ldots , {{\textbf{X}}}_J} = \max _{{{\textbf{a}}}_{11}, \ldots , {{\textbf{a}}}_{1 p_1} \ldots {{\textbf{a}}}_{J 1} \ldots {{\textbf{a}}}_{J p_J}} \displaystyle \sum _{j,k=1 ; j \not = k}^{J} c_{jk} g \left[ \sum _{l=1}^{p_j} \sum _{m=1}^{p_k} \frac{1}{n} {{\textbf{a}}}_{jl}^T \widehat{{{\textbf{K}}}}_{jl}^T \widehat{{{\textbf{K}}}}_{km} {{\textbf{a}}}_{km} \right] \end{aligned}$$

subject to $(1-\tau _j) \sum _{l=1}^{p_j} \sum _{l'=1}^{p_j} \frac{1}{n} {{\textbf{a}}}_{jl}^T \widehat{{{\textbf{K}}}}_{jl}^T \widehat{{{\textbf{K}}}}_{jl'} {{\textbf{a}}}_{jl'} + \tau _j \sum _{l=1}^{p_j} {{\textbf{a}}}_{jl}^T \widehat{{{\textbf{K}}}}_{jl} {{\textbf{a}}}_{jl} = 1,$ for $j=1, \ldots , J.$

To find the solution, $\left\{ \widehat{{{\textbf{a}}}}_{11}, \ldots , \widehat{{{\textbf{a}}}}_{1 p_1}, \ldots , \widehat{{{\textbf{a}}}}_{J 1}, \ldots , \widehat{{{\textbf{a}}}}_{J p_J} \right\}$ to the equation (7), an algorithm similar to the one considered in Tenenhaus et al.^13^ is developed. The detailed algorithm is described in the Supplementary Appendix A.

In the classical CCA method, the contribution of a variable in a group in relation between the group and the other group is measured by correlation^23^. To be specific, the contribution of $X_{1l}$ in ${{\textbf{X}}}_1$ for the relation between ${{\textbf{X}}}_1$ and ${{\textbf{X}}}_2$ is measured by $\mathrm {Corr}( {\widehat{b}}_{1l} X_{1l}, {\widehat{{{\textbf{b}}}}}_{2}^T {{\textbf{X}}}_2 )$, where ${\widehat{{{\textbf{b}}}}}_1$ and ${\widehat{{{\textbf{b}}}}}_2$ are canonical weights in CCA. A high absolute value of $\mathrm {Corr}( {\widehat{b}}_{1l} X_{1l}, {\widehat{{{\textbf{b}}}}}_{2}^T {{\textbf{X}}}_2 )$ implies that $X_{1l}$ plays a significant role in the relation between ${{\textbf{X}}}_1$ and ${{\textbf{X}}}_2$. Similarly, we can measure the contribution of a variable in a group in relation between the group and the other group in our approach, GAKCCA. We define the contribution coefficient of $X_{jl}$, the *l*th variable in the *j*th group, in relation between ${{\textbf{X}}}_j$ and ${{\textbf{X}}}_k$ as

$$\begin{aligned} r_{X_{jl},{{\textbf{X}}}_k}= & {} \mathrm {Corr}\Big ( f_{jl} (X_{jl}), f_{k} ({{\textbf{X}}}_{k}) \Big ). \end{aligned}$$

We also define the measure for the relation between ${{\textbf{X}}}_{j}$ and ${{\textbf{X}}}_k$ as

$$\begin{aligned} r_{{{\textbf{X}}}_j,{{\textbf{X}}}_k}= & {} \mathrm {Corr}\Big ( f_{j} ({{\textbf{X}}}_{j}), f_{k} ({{\textbf{X}}}_{k}) \Big ). \end{aligned}$$

The empirical version of $r_{X_{jl}, {{\textbf{X}}}_k}$ and $r_{{{\textbf{X}}}_j, {{\textbf{X}}}_k}$ can be formulated as

$$\begin{aligned} {\widehat{r}}_{X_{jl},{{\textbf{X}}}_k}= & {} {\widehat{\mathrm {Corr}}} \Big ( f_{jl} (X_{jl}), f_{k} ({{\textbf{X}}}_{k}) \Big ) = \displaystyle \frac{\displaystyle \sum _{m=1}^{p_k} \widehat{{{\textbf{a}}}}_{jl}^T \widehat{{{\textbf{K}}}}_{jl}^T \widehat{{{\textbf{K}}}}_{km} \widehat{{{\textbf{a}}}}_{km}}{\sqrt{\widehat{{{\textbf{a}}}}_{jl}^T \widehat{{{\textbf{K}}}}_{jl}^T \widehat{{{\textbf{K}}}}_{jl} \widehat{{{\textbf{a}}}}_{jl} }\, \sqrt{\displaystyle \sum _{m=1}^{p_k} \displaystyle \sum _{m'=1}^{p_k} \widehat{{{\textbf{a}}}}_{km}^T \widehat{{{\textbf{K}}}}_{km}^T \widehat{{{\textbf{K}}}}_{km'} \widehat{{{\textbf{a}}}}_{km'}} }, \end{aligned}$$

and

$$\begin{aligned} {\widehat{r}}_{{{\textbf{X}}}_j,{{\textbf{X}}}_k}= & {} \displaystyle \frac{\displaystyle \sum _{l=1}^{p_j} \sum _{m=1}^{p_k} \widehat{{{\textbf{a}}}}_{jl}^T \widehat{{{\textbf{K}}}}_{jl}^T \widehat{{{\textbf{K}}}}_{km} \widehat{{{\textbf{a}}}}_{km}}{\sqrt{\displaystyle \sum _{l=1}^{p_j} \displaystyle \sum _{l'=1}^{p_j} \widehat{{{\textbf{a}}}}_{jl}^T \widehat{{{\textbf{K}}}}_{jl}^T \widehat{{{\textbf{K}}}}_{jl'} \widehat{{{\textbf{a}}}}_{jl'} } \sqrt{ \displaystyle \sum _{m=1}^{p_k} \displaystyle \sum _{m'=1}^{p_k} \widehat{{{\textbf{a}}}}_{km}^T \widehat{{{\textbf{K}}}}_{km}^T \widehat{{{\textbf{K}}}}_{km'} \widehat{{{\textbf{a}}}}_{km'}} }. \end{aligned}$$

Simulation study shows that empirical contribution coefficient and measure for the relation between two groups describe structural information of variable groups well.

#### Regularization parameter selection

There can be several approaches for choosing appropriate regularization parameters. We consider a cross validation idea for selecting regularization parameters for GAKCCA. Using the whole data, we approximate $f_j$ and denote as ${\hat{f}}_j$. Using the split data, we approximate $f_j$ and denote as ${\hat{f}}_j^{-g}$ which is obtained by excluding the *g*th split. Then, we compare these two estimates to select the regularization parameters. This approach is similar to that of Ashad Alam and Fukumizu^24^.

In detail, we describe the selection procedure as follows. We split the *n* samples of $\left\{ {{\textbf{X}}}_1, \ldots , {{\textbf{X}}}_J \right\}$ into G subsets, denoting ${{\textbf{x}}}[1], \ldots , {{\textbf{x}}}[G]$, where ${{\textbf{x}}}[g]$ contains $n_g$ samples of $\left\{ {{\textbf{X}}}_1, \ldots , {{\textbf{X}}}_J \right\}$ and $n_1 + \ldots n_G = n$. For each $j = 1, \ldots , J$, we estimate $f_j$ such that

$$\begin{aligned} {\widehat{f}}_j= & {} \sum _{l=1}^{p_j} \sum _{i=1}^{n} {\widehat{a}}^{(i)}_{jl} \phi _{jl}^{(i)}, ~~~ {\widehat{f}}^{-g}_j = \sum _{l=1}^{p_j} \sum _{ i : X_{jl}^{(i)} \notin X[(g)] } {\widehat{a}}_{jl}^{(i),(-g)} \phi _{jl}^{(i),(-g)}, \end{aligned}$$

where ${\widehat{a}}_{jl}^{(i),(-g)}$ and $\phi _{jl}^{(i),(-g)}$ are calculated from the data excluding ${{\textbf{x}}}[g]$ while ${\widehat{a}}^{(i)}_{jl}$ and $\phi _{jl}^{(i)}$ are obtained from the entire data. Then, we obtain

$$\begin{aligned} L(\varvec{\tau }) = L(\tau _1, \ldots , \tau _J) = \frac{1}{G} \sum _{g=1}^{G} \sum _{j =1}^{J} \sum _{{{\textbf{x}}}\in {{\textbf{x}}}[g]} \left( \frac{{\widehat{f}}_j({{\textbf{x}}}) - {\widehat{f}}^{(-g)}_j({{\textbf{x}}})}{{\widehat{f}}_j({{\textbf{x}}})} \right) ^2 \end{aligned}$$

and selection of $\tau$ is made by minimizing $L(\varvec{\tau })$. The main idea of this procedure is that $f_{jl}$ can be expressed as $f_{jl} = \sum _{i=1}^{n} a_{jl}^{(i)} {\widehat{\phi }}_{jl}^{(i)} + f_{jl}^{perp}$ by reproducing property in RKHS and we consider $\sum _{i=1}^{n} a_{jl}^{(i)} {\widehat{\phi }}_{jl}^{(i)}$ as an approximation of $f_{jl}$. Then cross validation procedure chooses $\varvec{\tau } = (\tau _1, \ldots , \tau _J)$ which minimizes the variability of the estimate of $f_j$ caused by the selection of data. Note that $\tau _j$’s may not be equal, but for the purpose of simplicity in computation, we assume all $\tau _j$’s are equal in the simulation study and real data analysis.

#### Permutation test

In the classical CCA method, Wilks’ lambda statistic is widely used to test the hypothesis that there is no relationship between two groups^25^. However, it is difficult to apply the Wilks’ lambda test for GAKCCA due to multivariate normal distribution assumption of the Wilks’ lambda test. Nonlinear extension of GAKCCA makes the model more complex, so formulating test statistics based on the unknown nonlinear function is not feasible. Thus, we consider a permutation test approach to test $\rho _{{{\textbf{X}}}_1, \ldots , {{\textbf{X}}}_J} =0$. That is, we approximate the sampling distribution of test statistics, ${\widehat{\rho }}_{{{\textbf{X}}}_1, \ldots , {{\textbf{X}}}_J}$, by obtaining test statistics from resampling under the null hypothesis.

First, from the original data, we calculate ${\widehat{\rho }}_{{{\textbf{X}}}_1, \ldots , {{\textbf{X}}}_J}$, denoted as ${\widehat{\rho }}_{{{\textbf{X}}}_1, \ldots , {{\textbf{X}}}_J}^{obs}$. Second, for the *j*-th group, we sample $\left\{ {{\textbf{x}}}_j^{(1)*}, \ldots , {{\textbf{x}}}_j^{(n)*} \right\}$ from $\left\{ {{\textbf{x}}}_j^{(1)}, \ldots , {{\textbf{x}}}_j^{(n)} \right\}$ with replacement. We do the same procedure for all groups. Note that the resampled set $\left\{ {{\textbf{x}}}_1^{(k)*}, \ldots , {{\textbf{x}}}_J^{(k)*} \right\}$ does not necessarily keep the order as it should not matter under the null hypothesis. Third, from the resampled data, we calculate ${\widehat{\rho }}_{{{\textbf{X}}}_1, \ldots , {{\textbf{X}}}_J}$. Fourth, we repeat second and third steps *m* times and obtain ${\widehat{\rho }}_{{{\textbf{X}}}_1, \ldots , {{\textbf{X}}}_J}^{\left\{ 1 \right\} } \ldots , {\widehat{\rho }}_{{{\textbf{X}}}_1, \ldots , {{\textbf{X}}}_J}^{\left\{ m\right\} }$. Lastly, we find an empirical distribution ${\widehat{F}}$ from ${\widehat{\rho }}_{{{\textbf{X}}}_1, \cdots , {{\textbf{X}}}_J}^{\left\{ 1 \right\} } \cdots , {\widehat{\rho }}_{{{\textbf{X}}}_1, \cdots , {{\textbf{X}}}_J}^{\left\{ m\right\} }$. We reject the null hypothesis if $1- {\widehat{F}}\left( {\widehat{\rho }}_{{{\textbf{X}}}_1, \ldots , {{\textbf{X}}}_J}^{obs}\right)$ is less than the pre-specified significant level. In this paper, we set $m=300$.

Analogous hypothesis test methods can be applied to test whether a certain variable is helpful for relationship within groups or not via the contribution coefficient.

### Ethic approval

The data collection was approved by the Seoul National University Research Ethics Committee and all methods to collect the data were performed in accordance with the relevant guidelines and regulations. Informed written consent was obtained from all participants prior to actual participation. Also, all data were anonymized prior to analysis.

## Results

### Simulation study

To check the effectiveness of our method, we consider two synthesized data; one is an inter-independent case (Case I) and the other is an inter-dependent case (Case II).

For Case I, we consider 3 groups of variables (${{\textbf{X}}}_1$, ${{\textbf{X}}}_2$, ${{\textbf{X}}}_3$). The number of members in each group and their distribution assumption are as follows:

- $${{\textbf{X}}}_1 = (X_{11}, X_{12})^T$$ : $$X_{11} \sim N(0,1)$$, $$X_{12} \sim N(0,1)$$
- $${{\textbf{X}}}_2 = (X_{21}, X_{22}, X_{23}, X_{24})^T$$ : $$X_{21} \sim N(0,1)$$, $$X_{22} \sim N(0,1)$$, $$X_{23} \sim N(0,1)$$, $$X_{24} \sim N(0,1)$$
- $${{\textbf{X}}}_3 = (X_{31}, X_{32}, X_{33})^T$$ : $$X_{31} \sim N(0,1)$$, $$X_{32} \sim N(0,1)$$, $$X_{33} \sim N(0,1)$$

Here we assume that all *N*(0, 1)s are independent so that 3 groups ${{\textbf{X}}}_1,{{\textbf{X}}}_2$ and ${{\textbf{X}}}_3$ are mutually independent. From this setting, we generate 100 data points, that is, the number of samples is 100 ($n=100$).

To apply our method, GAKCCA, we use a Gaussian kernel for each variable. A Gaussian kernel for the *l*th variable in the *j*th block is given as $\phi _{jl} (x, y) = \exp \left( -\frac{||x-y||^2}{2 \sigma _{jl}^2} \right)$, where $\sigma _{jl}$ can be viewed as a bandwidth. We set $\sigma _{jl}$ by median distance between the data points in $\{ x_{jl}^{(1)}, \ldots , x_{jl}^{(n)} \}$ as in Balakrishnan et al.^14^ and Tenenhaus et al.^13^. We use a fully-connected design matrix, that is, $c_{jk} = 1$ if $j \not =k$ and $c_{jk}=0$, otherwise. We adopt a Horst scheme function, $g(x) = x$. Without further notice, Gaussian kernel with median-based bandwidth, fully-connected design matrix and Horst scheme function are used in all simulation study and real data analysis in this paper.

For the simulated data, we obtain estimates of $\rho _{{{\textbf{X}}}_1, {{\textbf{X}}}_2, {{\textbf{X}}}_3}$, $r_{{{\textbf{X}}}_1, {{\textbf{X}}}_2}$, $r_{{{\textbf{X}}}_2, {{\textbf{X}}}_3}$ and $r_{{{\textbf{X}}}_3, {{\textbf{X}}}_1}$. By the permutation test described in the previous section, we can calculate a p-value for testing each quantity being zero. We repeat this procedure using three hundreds sets of simulated data.

Table 1 shows that there is no significant relationship between 3 groups (p-value of ${\widehat{\rho }}_{{{\textbf{X}}}_1, {{\textbf{X}}}_2, {{\textbf{X}}}_3}$ is 0.517 on average), which correctly captures dependence/independence of the simulation setting for Case I.

**Table 1:** Averages of estimated values and the corresponding p-values from the permutation test over 300 simulated data sets for the Case I (Independent case). The number in the parenthesis is standard deviation over 300 simulated data sets.

	Estimate	p-value
$\rho _{{{\textbf{X}}}_1, {{\textbf{X}}}_2, {{\textbf{X}}}_3}$	0.544 (0.210)	0.517 (0.268)
$r_{{{\textbf{X}}}_1, {{\textbf{X}}}_2}$	0.303 (0.115)	0.447 (0.289)
$r_{{{\textbf{X}}}_2, {{\textbf{X}}}_3}$	0.341 (0.077)	0.465 (0.300)
$r_{{{\textbf{X}}}_3, {{\textbf{X}}}_1}$	0.287 (0.098)	0.421 (0.286)

For Case II, we consider 3 groups (${{\textbf{Y}}}_1$, ${{\textbf{Y}}}_2$, ${{\textbf{Y}}}_3$) again and the number of members in each group and their distribution assumption are as follows.

- $${{\textbf{Y}}}_1 = (Y_{11}, Y_{12})^T$$ : $$Y_{11} \sim z + N(0,1)$$, $$Y_{12} \sim N(0,1)$$
- $${{\textbf{Y}}}_2 = (Y_{21}, Y_{22}, Y_{23}, Y_{24})^T$$ : $$Y_{21} \sim N(0,1)$$, $$Y_{22} \sim z^2 + N(0,1)$$, $$Y_{23} \sim N(0,1)$$, $$Y_{24} \sim N(0,1)$$
- $${{\textbf{Y}}}_3 = (Y_{31}, Y_{32}, Y_{33})^T$$ : $$Y_{31} \sim |z| + N(0,1)$$, $$Y_{32} \sim z \sin (z) + N(0,1)$$, $$Y_{33} \sim N(0,1)$$,

where *z* follows $\text {uniform} [-5, 5]$. Here we assume all *N*(0, 1)s are independent. Given the structure of the groups, $Y_{11}$, $Y_{22}$, $Y_{31}$ and $Y_{32}$ are linked with nonlinear relationship.

From this setting, we generate 100 data points, that is, the number of samples is 100 ($n=100$) and apply our method to estimate $\rho _{{{\textbf{Y}}}_1, {{\textbf{Y}}}_2, {{\textbf{Y}}}_3}$, $r_{{{\textbf{Y}}}_1, {{\textbf{Y}}}_2}$, $r_{{{\textbf{Y}}}_2, {{\textbf{Y}}}_3}$ and $r_{{{\textbf{Y}}}_3, {{\textbf{Y}}}_1}$. We also obtain the corresponding p-values by the permutation test. We repeat this procedure with 300 simulated data sets. The averages of estimated values and the p-values are provided in Table  2. A small p-value for testing $\rho _{{{\textbf{Y}}}_1, {{\textbf{Y}}}_2, {{\textbf{Y}}}_3}=0$ indicates that the groups are related. We can also see from small p-values of $r_{{{\textbf{Y}}}_1, {{\textbf{Y}}}_2}$, $r_{{{\textbf{Y}}}_2, {{\textbf{Y}}}_3}$ and $r_{{{\textbf{Y}}}_3, {{\textbf{Y}}}_1}$ that all three groups are inter-related, which implies that our approach capture dependence between groups correctly for Case II. Note that the value of $\rho _{{{\textbf{Y}}}_1, {{\textbf{Y}}}_2, {{\textbf{Y}}}_3}$ can be larger than one as it is a combination of functions of covariances. On the other hand, the relation measure $r_{{{\textbf{Y}}}_j, {{\textbf{Y}}}_k}$ should be less than equal to one as it is a correlation. Also note that 0.000 in the Table 2. indicates the value is zero when it is rounded to the nearest thousandth.

**Table 2:** Averages of estimated values and the corresponding p-values from the permutation test over 300 simulated data sets for the Case II (dependent case). The number in parenthesis is standard deviation over 300 simulated data sets.

	Estimate	p-value
$\rho _{{{\textbf{Y}}}_1, {{\textbf{Y}}}_2, {{\textbf{Y}}}_3}$	1.992 (0.419)	0.000 (0.001)
$r_{{{\textbf{Y}}}_1, {{\textbf{Y}}}_2}$	0.779 (0.044)	0.000 (0.000)
$r_{{{\textbf{Y}}}_2, {{\textbf{Y}}}_3}$	0.911 (0.022)	0.000 (0.000)
$r_{{{\textbf{Y}}}_3, {{\textbf{Y}}}_1}$	0.728 (0.051)	0.000 (0.000)

To investigate which variable in the group contributes to the relationship, we calculate contribution coefficients, $r_{Y_{jl}, {{\textbf{Y}}}_k}$ introduced in the previous section. The results are given in Table 3. Recall that $Y_{11}$, $Y_{22}$, $Y_{31}$ and $Y_{32}$ have a common component *z* in the simulation setting. The bold letters in the first column of Table  3 indicate this true relationship while the bold numbers in the second to fourth columns indicates small p-value cases. $Y_{11}$ in the first group ${{\textbf{Y}}}_1$ is the one that contributes to the relation between ${{\textbf{Y}}}_1$ and ${{\textbf{Y}}}_2$, and between ${{\textbf{Y}}}_1$ and ${{\textbf{Y}}}_3$. The empirical contribution coefficients and the corresponding p-values show that $Y_{11}$ is contributing to that relationship compared to $Y_{12}$. Similarly, we can see from Table 3 that the empirical contribution coefficients successfully capture the contribution of $Y_{22}$, $Y_{31}$ and $Y_{32}$ in relation between their corresponding group and the other groups.

**Table 3:**
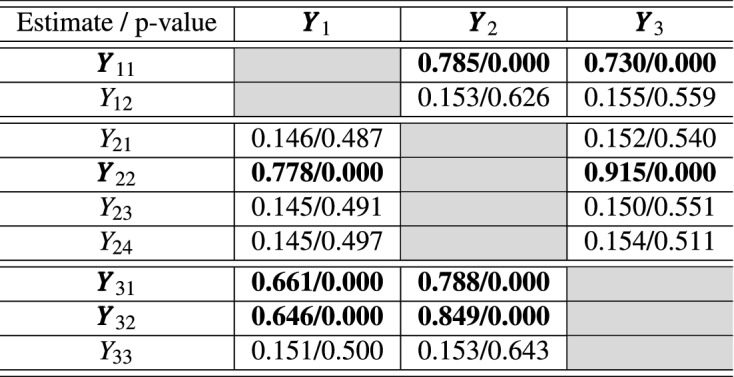
Averages of empirical contribution coefficients and the corresponding p-values from the permutation test over 300 simulated data sets for the Case II (dependent case).

**Figure 1:**
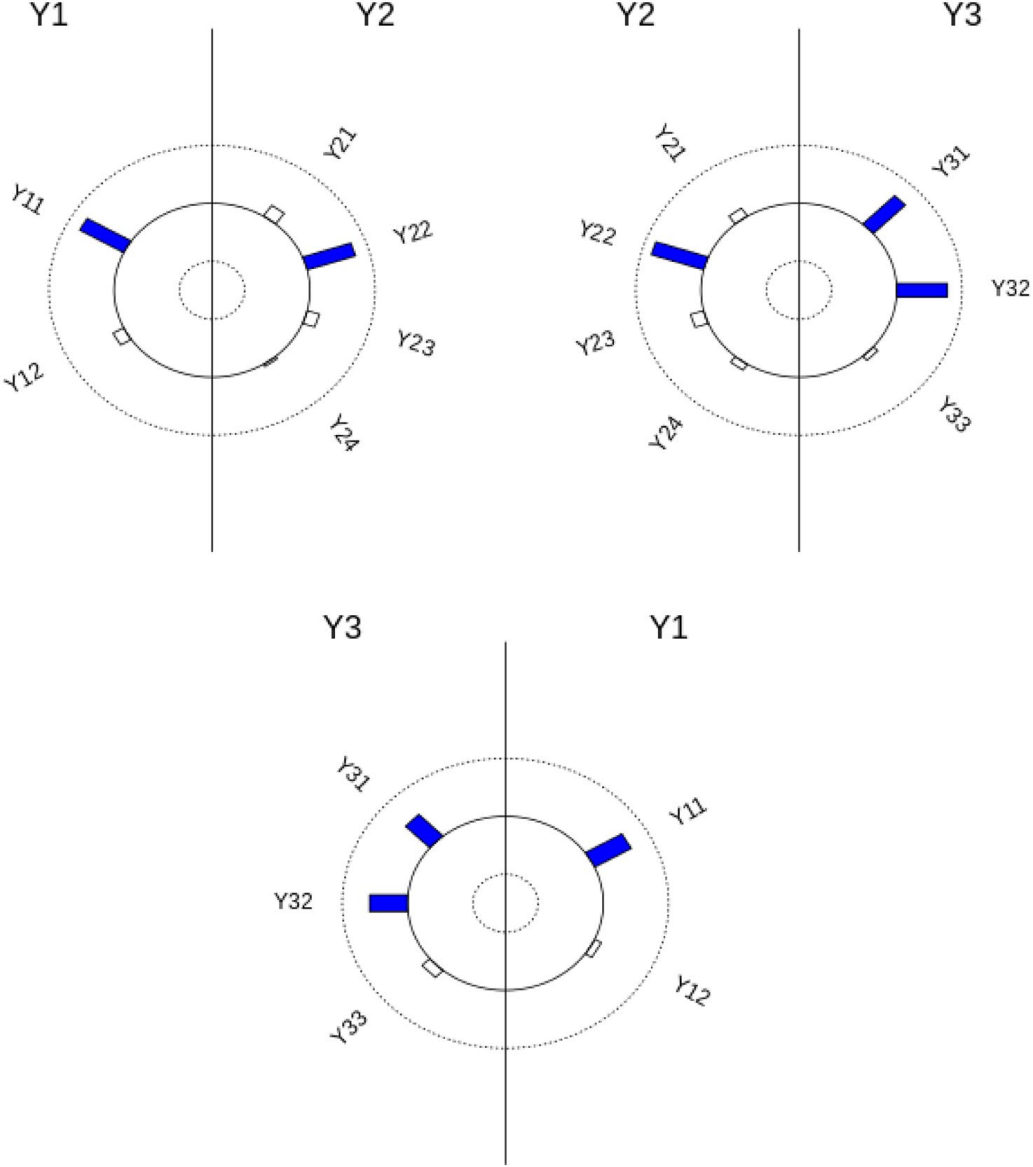
Helio plots of contribution coefficients $r_{Y_{jl}, {{\textbf{Y}}}_k}$ in Case II. The size of a bar indicates the value of empirical contribution coefficient of that variable to the other group. Blue colored bars means the p-value of the corresponding empirical contribution coefficient is below 0.05.

To visualize contribution of each variable in relation with the other group, we utilize a helio plot. Figure 1 shows helio plots between pairs of groups in the second simulation setting (Case II). In the helio plot, variables in two groups are listed in a circular layout. The size of a bar indicates the value of empirical contribution coefficient of that variable to the other group. For example, in the upper left helio plot in Fig. 1, the size of the bar corresponding to $Y_{11}$ represents the value of empirical contribution coefficient of $Y_{11}$ to ${{\textbf{Y}}}_2$, i.e. ${\widehat{r}}_{Y_{11}, {{\textbf{Y}}}_{2}}$. Also, blue colored bars means the p-value of the corresponding empirical contribution coefficient is below 0.05. Thus, $Y_{11}$ has a significant influence on the relation to ${{\textbf{Y}}}_2$ and $Y_{12}$ is less relevance in the relation to ${{\textbf{Y}}}_{2}$ when we set 0.05 as the significance level. Similarly, from the same helio plot, $Y_{22}$ has a significant influence on the relation to ${{\textbf{Y}}}_1$ and the other variables in ${{\textbf{Y}}}_{2}$ except $Y_{22}$ are less relevance in relation to ${{\textbf{Y}}}_{1}$. From Fig. 1, we can see that GAKCCA reveals nonlinear relation between groups and contribution in Case II, properly.

**Table 4:** Averages of estimated absolute values and the corresponding p-values from the permutation test over 300 simulated data for Case II (dependent case) based on RGCCA model. The number in parentheses is standard deviation over 300 simulated data.

	Estimate	p-value
$|\rho _{{{\textbf{Y}}}_1, {{\textbf{Y}}}_2, {{\textbf{Y}}}_3}|$	1.299 (0.198)	0.000 (0.002)
$|r_{{{\textbf{Y}}}_1, {{\textbf{Y}}}_2}|$	0.164 (0.073)	0.518 (0.276)
$|r_{{{\textbf{Y}}}_2, {{\textbf{Y}}}_3}|$	0.875 (0.024)	0.000 (0.000)
$|r_{{{\textbf{Y}}}_3, {{\textbf{Y}}}_1}|$	0.164 (0.072)	0.579 (0.288)

**Table 5:** Averages of empirical absolute contribution coefficients and the corresponding p-values from the permutation test over 300 simulated data for Case II (dependent case) based on RGCCA model.

Estimate/p-value	${{\textbf{Y}}}_1$	${{\textbf{Y}}}_2$	${{\textbf{Y}}}_3$
${{\textbf{Y}}}_{11}$		0.121/0.500	0.117/0.519
$Y_{12}$		0.094/0.588	0.094/0.610
$Y_{21}$	0.085/0.532		0.083/0.578
${{\textbf{Y}}}_{22}$	0.140/0.308		0.890/0.000
$Y_{23}$	0.085/0.529		0.083/0.574
$Y_{24}$	0.087/0.517		0.080/0.594
${{\textbf{Y}}}_{31}$	0.124/0.411	0.774/0.000	
${{\textbf{Y}}}_{32}$	0.135/0.385	0.790/0.000	
$Y_{33}$	0.084/0.568	0.088/0.619	

We applied RGCCA to the simulated data of Case II (dependent case) for the comparison with GAKCCA. We utilized RGCCA package in **R** (www.r-project.org) and implemented the permutation test to extract significant groups. The design matrix, scheme function, the number of resamples for the permutation test and the number of simulated data sets are same as the ones that we considered for GAKCCA. In applying RGCCA, the sign of coefficients changed frequently during the respective simulation and permutation test, so the absolute value of coefficients was considered when we summarized the results. The results are given in Tables 4 and  5. The RGCCA result shows that there is a significant relationship between $Y_2$ and $Y_3$ (The average of absolute value of empirical correlation between first canonical variate of $Y_2$ and that of $Y_3$ is 0.875 with p-value 0.000), but weak relationship between $Y_1$ and $Y_2$, and between $Y_1$ and $Y_3$ compared to the results from GAKCCA (The averages of empirical correlations from RGCCA are 0.164, 0.164 with p-value 0.518, 0.579, respectively). The limitation of RGCCA that can only consider linear relationship between groups leads to a failure in identifying clear nonlinear relationship within them.

### Real data application

We used the data on individuals’ measures such as demographic information, a number of psychometric questionnaires as well as structural MRI. The data were from 86 undergraduate students in Seoul National University, Seoul, Korea. We analyzed these data using GAKCCA to find out the relationship between four domains (Neurodevelopmental, Psychosocial, Clinical and Neurophysiological domains). A full list of variables in each domain is available in Supplementary Table S6 in the Online Appendix B. Six participants who had high level of Beck Depression Inventory (BDI-II) or Beck Anxiety Inventory (BAI) were excluded so that we use measurements from 80 participants ($n=80$). To apply GAKCCA method to these data, we chose fully connected design matrix, Gaussian kernel with median-based bandwidth and the Horst scheme function. Also, we set the number of samples for the permutation test to 8,000 ($m=8,000$).

When we first applied GAKCCA to this data, we found that the significant association between domains as follows: neurodevelopmental and neurophysiological domains, psychosocial and clinical domains, and clinical and neurophysiological domains. According to this initial finding, the design matrix was modified to maintain the relationships within relevant domains while eliminating those within irrelevant domains in order to clarify the between-group structure. The GAKCCA model was applied with the new design matrix again.

Consistent with previous studies investigating the structural brain correlates of IQ^26,27^, we defined that there are significant relationships between the neurodevelopmental and the neurophysiological domains (Empirical contribution coefficient is 0.463 with p-value 0.048). Also, a trend toward significance (p = 0.077) is also reported in the clinical and neurophysiological domains (Empirical contribution coefficient is 0.463). In both results, the T1 volume data from structural MRI in the neurophysiological domain appeared to play the most dominant role in association to the neurodevelopmental domain and clinical domain, respectively (Fig. 3).

On the other hand, the alcohol use disorder (AUDIT-K) and anxiety (BAI) in the clinical domain shows the most dominant roles for the association to the neurophysiological domain. Also, IQ in the neurodevelopmental domain plays the most dominant role in association to the neurophysiological domain. In terms of this trend-level result, this seems quite plausible in that the current clinical domain was defined through the self-reported questionnaires, not having any diagnoses of psychiatric illnesses. Further research narrowing down the definition of the clinical domain is necessary to exclude individuals with subclinical symptoms.

**Figure 2:**
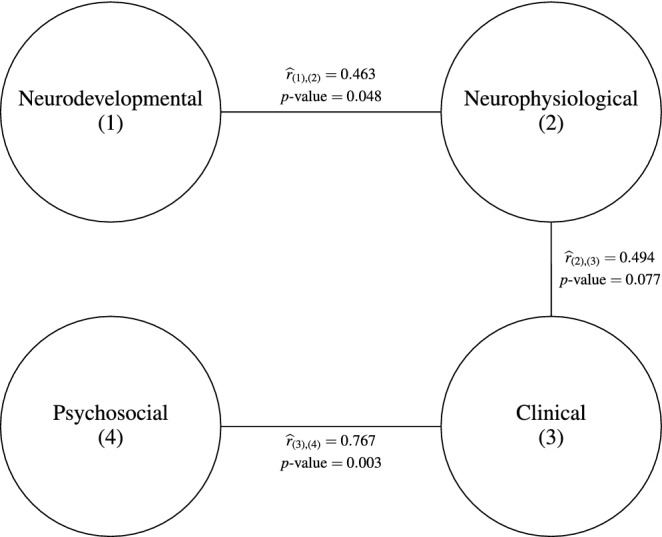
The diagram of the significant relationships between domains based on the GAKCCA model. ${\hat{r}}_{(i),(j)}$ values are empirical contribution coefficient between (*i*) and (*j*) domains, which is provided with p-values.

**Figure 3:**
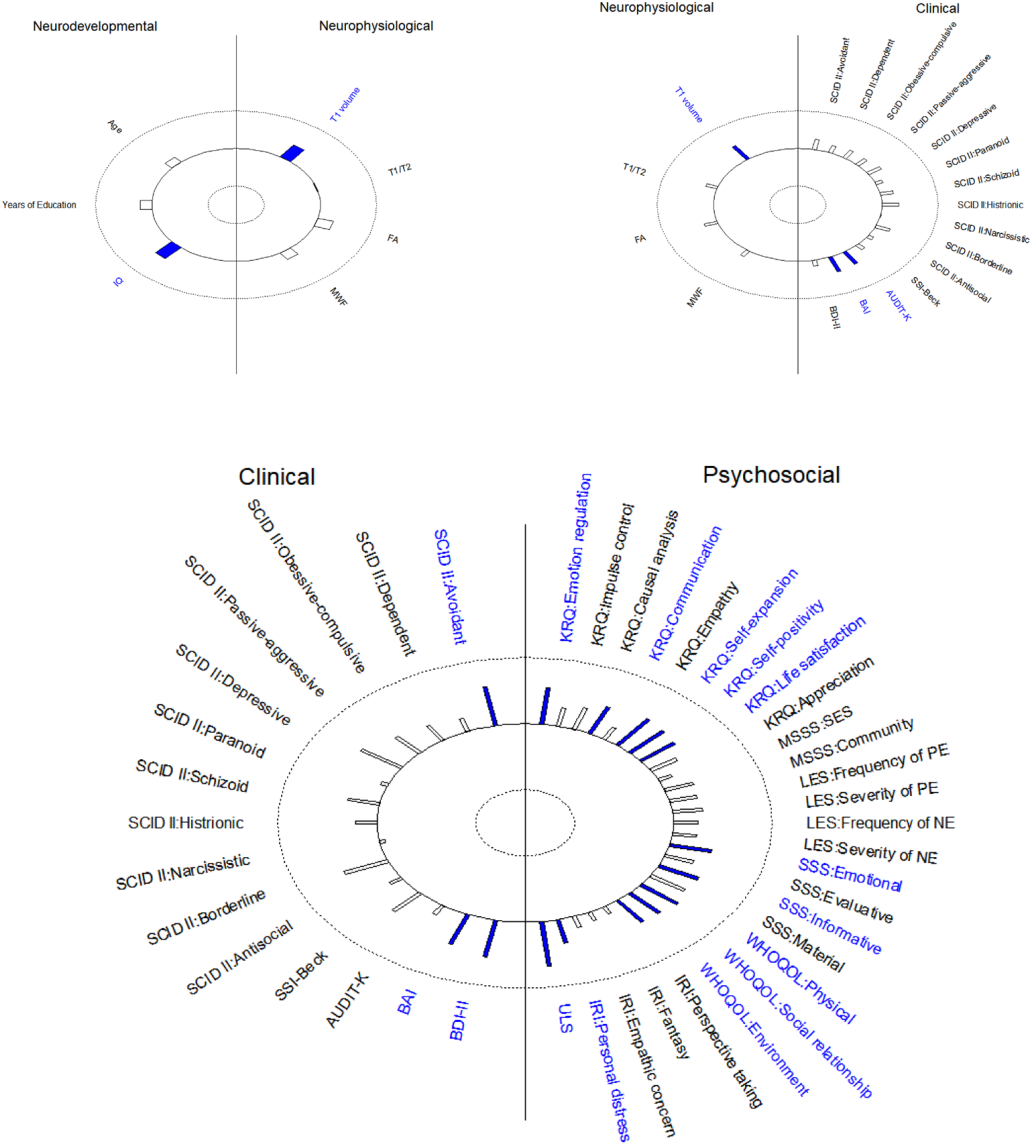
Helio plots of significant relationships based on GAKCCA model.

As expected, there was also a statistically significant relationship with a significance level 0.05 between the psychosocial domain and the clinical domain (Fig. 2). This finding was based on the empirical contribution coefficient of the psychosocial domain to the clinical domain (0.767 with p-value 0.003). With significance level 0.05, major variables contributing to the relationship are KRQ:Emotional regulation, KRQ:Communication, KRQ:Self-expansion, KRQ:Self-positivity, KRQ:Life satisfaction, SSS:Emotional support, SSS:Information support, WHOQOL:Physical, WHOQOL:Social relationship, WHOQOL:Environment, IRI:Personal distress and ULS in the psychosocial group (12 variables), and SCID II:Avoidant, BAI and BDI-II in the clinical group (3 variables) (Fig. 3). Specifically, the psychosocial domain reflecting psychological and environmental resources (KRQ, SSS, etc.) were found to be highly associated with the clinical domain, which was characterized by increased avoidant personality traits, anxiety, and depression. These findings are consistent with previous research^28–31^.

We also applied RGCCA to the data for comparison with GAKCCA. The design matrix, the scheme function and the number of samples for the permutation test are the same as the ones that we considered for GAKCCA. The RGCCA result shows that there is a significant relationship between psychosocial and clinical domains (The empirical correlation between first canonical variate of psychosocial domain and that of clinical domain is 0.779 with p-value 0.000), but more weak relationship between neurodevelopmental and neurophysiological domains, and clinical and neurophysiological domains than those from GAKCCA (The empirical correlations from RGCCA are 0.305 and 0.389 with p-value 0.320 and 0.124, respectively).

## Discussion

In this paper, we have proposed a generalized version of additive kernel CCA. Due to the nature of the objective function, the set of regularization parameters is introduced and we consider the cross validation by comparing estimated additive components for the selection of regularization parameters. A permutation-based test is introduced for checking the relationship between groups. Simulation study shows the proposed method can successfully identify nonlinear relationship between groups and reveals the influence of each variable in the group. Such advantages will be useful in many research areas that deal with multivariate data. However, the proposed approach may not properly handle applications where interactions between different variables in each group exist due to the assumption of additivity.

Compared with the classical CCA, which uses a simple test statistic such as Wilks’ lambda, permutation test requires more computation time. However, the computation burden can be effectively reduced by distributed computing. On the other hand, in selecting regularization parameters in GAKCCA, intensive computation is inevitable. Thus, it is worth investigating on developing an algorithm to make computation faster or finding a computationally more efficient selection method.

The classical CCA can consider the second canonical variates that maximize the correlation $\mathrm {Corr}\left( b_1^{T} X_1, b_2^{T} X_2 \right)$ among all choices that are uncorrelated with the first canonical variates. This is not straightforward in GAKCCA but it is worth investigating as a future research since it could reveal additional structural information within groups that the current GAKCCA model does not explain

GAKCCA on investigating relation among individuals’ measures that are categorized as one of neurodevelopmental, psychosocial, clinical and neurophysiological domains reveals more relationships than RGCCA and those findings are consistent with previous research.

## Supplementary information

Supplementary information
