# Supplementary material for: Multi-group analysis using generalized additive kernel canonical correlation analysis
Source: Sci Rep. 2020 Jul 28;10:12624. doi: 10.1038/s41598-020-69575-x (PMC7387565; doi:10.1038/s41598-020-69575-x)
Supplement: Supplementary file 1 — Supplementary information [file 41598_2020_69575_MOESM1_ESM.pdf]

Appendix A: Algorithm for GAKCCA

Algorithm 1 Generalized Additive Kernel Canonical Correlation Analysis

Choose arbitrary vectors $\tilde{\boldsymbol{a}}_{jl'}$ such that below condition holds :

$$\boldsymbol{a}^0_{jl'} = \left[(1-\tau_j)\frac{1}{n}\sum_{l=1}^{p_j}\sum_{m=1}^{p_j}\tilde{\boldsymbol{a}}^{0T}_{jl}\widehat{\boldsymbol{K}}_{jl}\widehat{\boldsymbol{K}}_{jm}\tilde{\boldsymbol{a}}^0_{jm} + \tau_j\sum_{l=1}^{p_j}\tilde{\boldsymbol{a}}^{0T}_{jl}\widehat{\boldsymbol{K}}_{jl}\tilde{\boldsymbol{a}}^0_{jl}\right]^{(-1/2)} \tilde{\boldsymbol{a}}^0_{jl'}$$

repeat

for $j \leftarrow 1$ to J **do**

for $l' \leftarrow 1$ to p_j **do**

$$\begin{aligned}
\boldsymbol{u}_j^{(s)} &= \sum_{l=1}^{l'-1}\widehat{\boldsymbol{K}}_{jl}\boldsymbol{a}_{jl}^{(s+1)} + \sum_{l=l'}^{p_j}\widehat{\boldsymbol{K}}_{jl}\boldsymbol{a}_{jl}^{(s)} \\
\boldsymbol{z}_j^{(s)} &= \sum_{k=1}^{j-1} c_{jk}w\left(\sum_{l=1}^{l'-1}\frac{1}{n}\boldsymbol{a}_{jl}^{T\,(s+1)}\widehat{\boldsymbol{K}}_{jl}\boldsymbol{u}_k^{(s+1)} + \sum_{l=l'}^{p_j}\frac{1}{n}\boldsymbol{a}_{jl}^{T\,(s)}\widehat{\boldsymbol{K}}_{jl}\boldsymbol{u}_k^{(s+1)}\right)\frac{1}{n}\boldsymbol{u}_k^{(s+1)} \\
&+ \sum_{k=j+1}^{J} c_{jk}w\left(\sum_{l=1}^{l'-1}\frac{1}{n}\boldsymbol{a}_{jl}^{T\,(s+1)}\widehat{\boldsymbol{K}}_{jl}\boldsymbol{u}_k^{(s)} + \sum_{l=l'}^{p_j}\frac{1}{n}\boldsymbol{a}_{jl}^{T\,(s)}\widehat{\boldsymbol{K}}_{jl}\boldsymbol{u}_k^{(s)}\right)\frac{1}{n}\boldsymbol{u}_k^{(s)} \\
r_j^{(s)} &= \sum_{l=1}^{l'-1}\boldsymbol{z}_j^{T\,(s+1)}\widehat{\boldsymbol{K}}_{jl}\boldsymbol{z}_j^{(s+1)} + \sum_{l=l'}^{p_j}\boldsymbol{z}_j^{T\,(s)}\widehat{\boldsymbol{K}}_{jl}\boldsymbol{z}_j^{(s)} \\
s_j^{(s)} &= \sum_{l=1}^{l'-1}\boldsymbol{u}_j^{T\,(s+1)}\widehat{\boldsymbol{K}}_{jl}\boldsymbol{z}_j^{(s+1)} + \sum_{l=l'}^{p_j}\boldsymbol{u}_j^{T\,(s)}\widehat{\boldsymbol{K}}_{jl}\boldsymbol{z}_j^{(s)} \\
\lambda_j &= -\left[\frac{1-\tau_j}{\tau_j}\frac{1}{n}s_j\right] \pm \sqrt{\left[\frac{1-\tau_j}{\tau_j}\frac{1}{n}s_j\right]^2 + 4\frac{1}{\tau_j}r_j} \\
\boldsymbol{a}_{jl'}^{(s+1)} &= \frac{1}{\tau_j}\frac{1}{\lambda_j^{(s)}}\boldsymbol{z}_j^{(s)} - \frac{1}{n}\frac{1-\tau_j}{\tau_j}\boldsymbol{u}_j^{(s)}
\end{aligned}$$

end for

end for

until convergence of the criterion;

Appendix B: List of variables in real data

Table 6. Variables and Definitions

Groups	Variables	Definition
Psychosocial group	KRQ:Emotion regulation	Korean Resilience Quotient (3 categories. 9 subscales): Self-Regulation, Master, Positivity<br>- Self-Regulation = Emotion Regulation + Impulse Control + Causal Analysis<br>- Interpersonal Skills = Communication Skills + Empathy + Reaching Out<br>- Positivity = Realistic Optimism + Life-Satisfaction + Gratitude
	KRQ:Impulse control	
	KRQ:Causal analysis	
	KRQ:Communication	
	KRQ:Empathy	
	KRQ:Self-expansion	
	KRQ:Self-positivity	
	KRQ:Life satisfaction	
	KRQ:Appreciation	
	MSSS:SES	MacArthur Scale of Subjective Social Status
	MSSS:Community	
	LES:Frequency of PE	Life Experiences Survey:<br>Frequency of Life Events, Severity of Life events, Intensity of Life Events
	LES:Severity of PE	
	LES:Frequency of NE	
	LES:Severity of NE	
	SSS:Emotional support	Attributes of Social Support: :<br>Emotional, Instrumental, Informational, and Appraisal
	SSS:Evaluative	
	SSS:Information support	
	SSS:Material	
	WHOQOL:Physical	Physical Health, Psychological Health, Social Relationships and Environment
	WHOQOL:Social relationship	
	WHOQOL:Environment	
	IRI:Perspective taking	Perspective Taking (PT), Fantasy (FS),<br>Empathic Concern (EC), and Personal Distress (PD)
	IRI:Fantasy	
	IRI:Empathic concern	
	IRI:Personal distress	
	ULS	Scale for Loneliness
Clinical group	SCIDII:Avoidant	Self-Questionnaire: Personality Disorders<br>- Cluster A (odd) -Paranoid, Schizoid, Schizotypal<br>- Cluster B (dramatic)- Antisocial, Borderline, Histrionic, Narcissistic<br>- Cluster C (anxious)- Avoidant, Dependent, Obsessive–compulsive
	SCIDII:Dependent	
	SCIDII:Obessive-compulsive	
	SCIDII:Passive-aggressive	
	SCIDII:Depressive	
	SCIDII:Paranoid	
	SCIDII:Schizoid	
	SCIDII:Histrionic	
	SCIDII:Narcissistic	
	SCIDII:Borderline	
	SCIDII:Antisocial	
	SSI-Beck	Scale for Suicide Ideation
	AUDIT-K	Scale for Alcohol Use Disorder
	BAI	Scale for Anxiety
	BDI-II	Scale for Depression
Structural MRI group	T1 volume	T1 - T2 weighted images
	T1/T2	T1 - T2 weighted images<br>(Indicator that indirectly shows the amount of myelin in the cortical grating meter.)
	FA	DTI-Fractional Anisotropy
	MWF	Myelin Water Fraction, ViSTA
Neurodevelopmental group	Age	Physical age
	Years of Education	Total training period in schools
	IQ	Estimated IQ
